# Case Report: Congenital Coronary Artery Ring With Single Left Coronary Ostium and Fistula: A Previously Unreported Anatomy

**DOI:** 10.3389/fcvm.2021.699529

**Published:** 2021-08-27

**Authors:** Shiyuan Tang, Mi Tang, Chukwuemeka Daniel Iroegbu, Jinfu Yang, Chengming Fan

**Affiliations:** Department of the Cardiovascular Surgery, The Second Xiangya Hospital, Central South University, Changsha, China

**Keywords:** coronary artery anomalies, single coronary ostium, giant coronary trunk, coronary artery fistula, coronary artery ring

## Abstract

**Background:** Single coronary ostium concomitant with coronary artery fistula is a very rare congenital anomaly. Apart from that, the combination of a closed loop of the coronary artery has never been reported.

**Case presentation:** Herein, we present a 7-year-old girl diagnosed as single left coronary ostium with a giant coronary trunk, coronary artery to right ventricle fistula, and coronary artery ring. The coronary fistula was surgically ligated with off-pump strategy and the patient discharged on postoperative day 5 and free of symptoms during the 3 years of follow-up.

**Conclusion:** To our knowledge, the presented congenital coronary anomaly is the first to be reported in the literature with the name of congenital coronary artery ring with single left coronary ostium and fistula.

## Background

Single coronary artery (SCA) in which one coronary artery emerges from a single coronary ostium in the aorta, is a very rare angiographic feature in congenital coronary artery abnormalities (0.024 to 0.066%), with different subtypes depending on the course of the abnormal artery (1). SCA can be either an isolated congenital cardiac disease or be associated with other congenital abnormalities. SCA combined with coronary artery fistula (CAF) is very rare and few cases have been reported (2–5). It's worth noting that SCA combined with CAF and coronary artery ring (left and right main coronary artery communicate with each other and form a closed loop) is extremely rare and easily misdiagnosed. Here we report a rare case of a SCA originates from the right aortic sinus, forming a giant coronary trunk, and associated with CAF with right ventricle (RV). Furthermore, the right main coronary artery was connected with the left conus branch via the collateral branch while the left circumflex was communicated with the right coronary artery thus forming a closed loop (CAR as we defined). The anomalies were successfully corrected with operation in our hospital. The case is first reported as we reviewed the literature.

## Case Presentation

**Figure 1:**
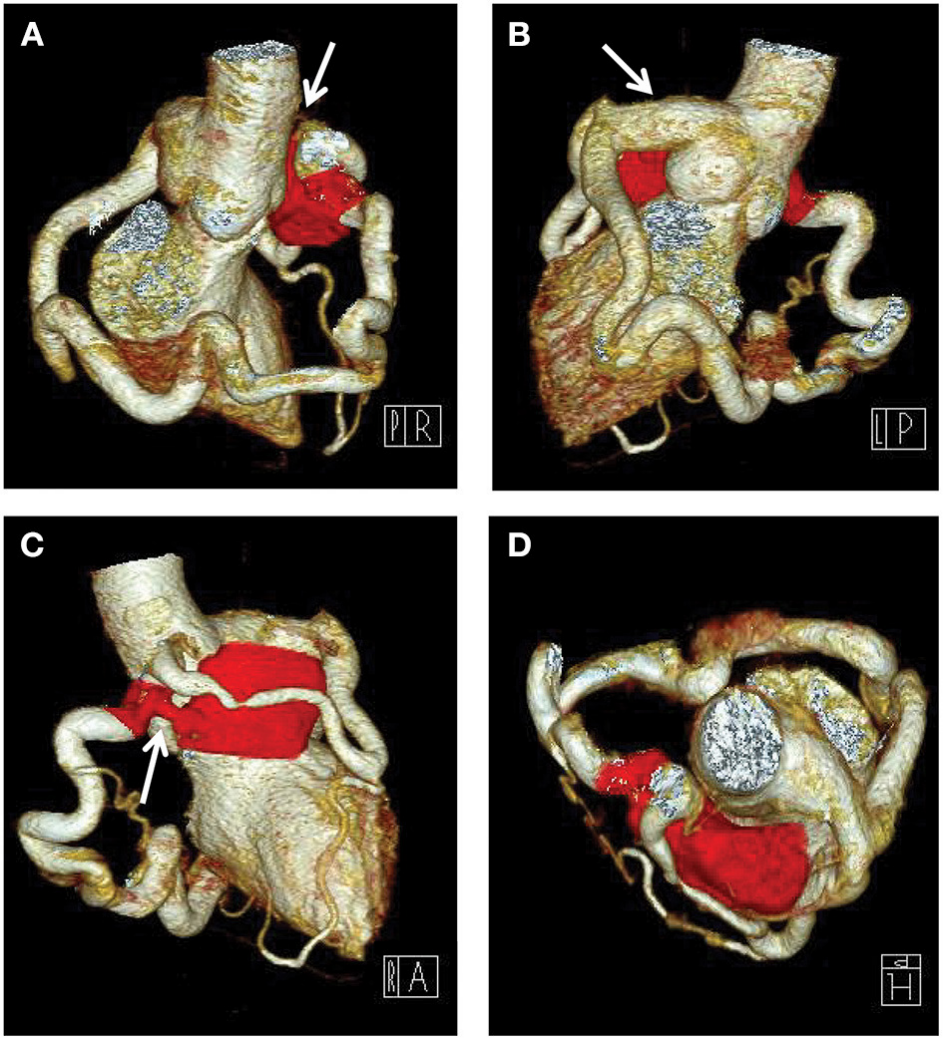
Computed tomography angiography preoperatively showing that there is an interruption between right sinus of Valsalva and the enlarged and tortuous right main coronary artery (**A**, arrow), a single left coronary ostium with a giant coronary trunk (**B**, arrow), a 7-mm fistula into the right ventricle (Red) (**C**, arrow), the left and right coronary artery were connected with each other and formed a closed loop at the base of the heart **(D)**.

**Figure 2:**
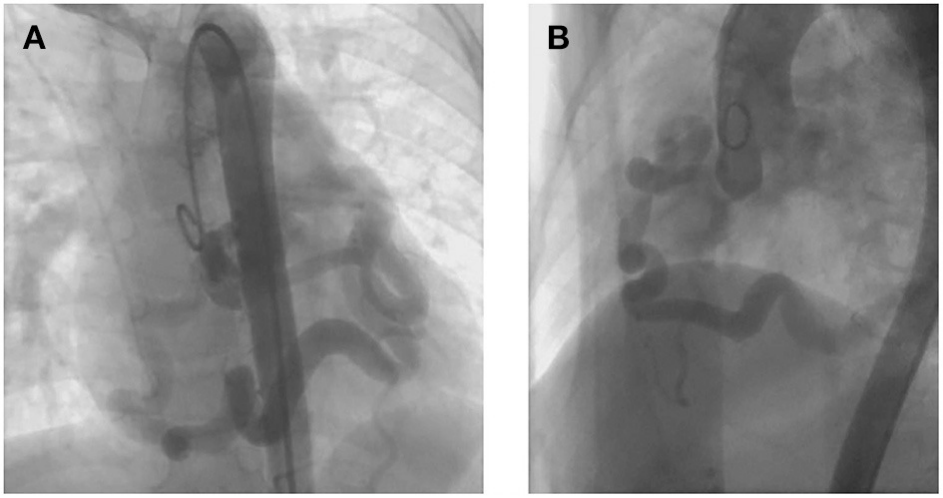
Ascending aortic angiography preoperatively showing that the left and right coronary artery were enlarged and tortuous **(A)**, right coronary artery was detected secondly to the left one **(B)**, confirming the diagnosis of coronary artery ring with single left coronary ostium and fistula.

**Figure 3:**
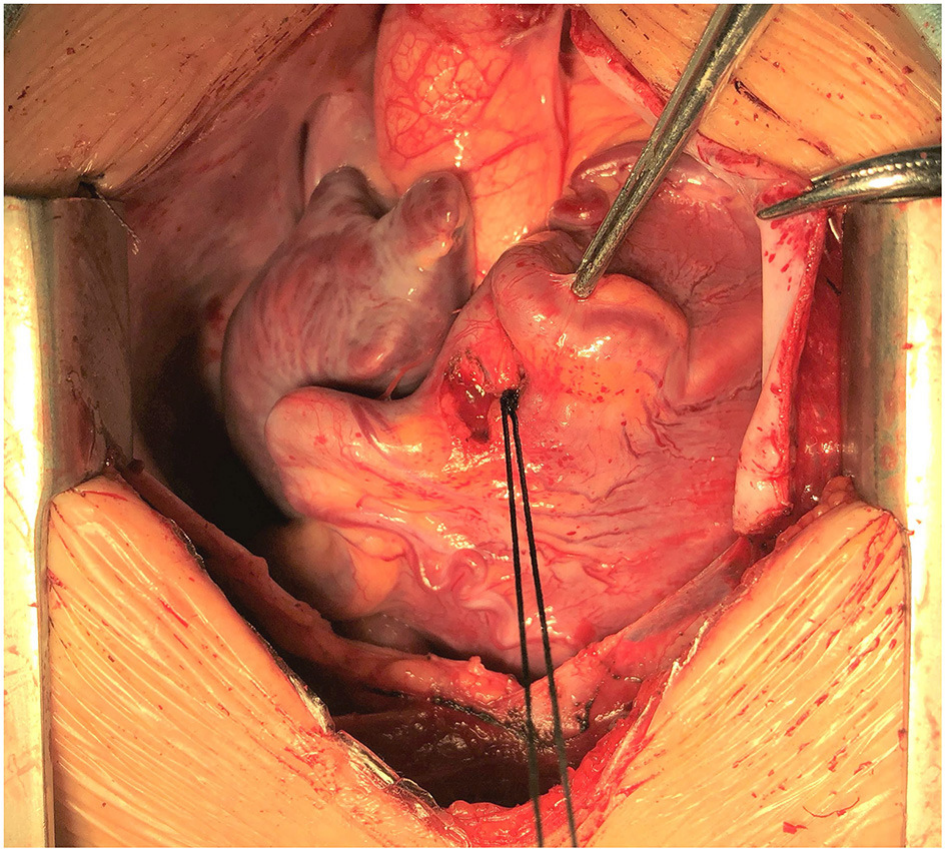
Intraoperative view showing that the fistula was ligated with off-pump strategy.

**Figure 4:**
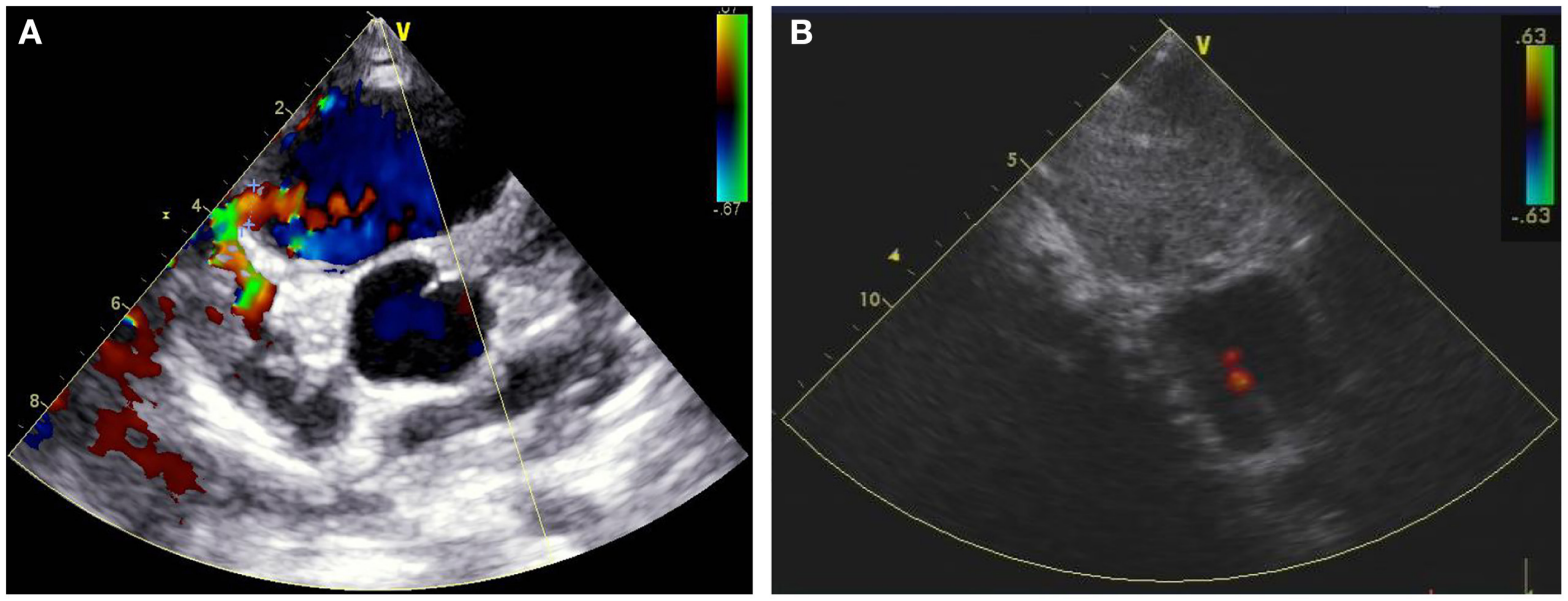
Transthoracic echocardiography showing that a 6.28-mm coronary fistula into the right ventricle (**A**, arrow) was detected preoperatively, and undetectable 1 week post-surgical ligation **(B)**.

A 7-year old girl with exertive chest tightness was referred to our institution. Physical examination was notable for a grade 3/6 systolic and diastolic cardiac murmur over the third left intercostal space. There was no other notable clinical findings during physical examination or medical history and no family history of cardiovascular disease. Electrocardiogram, Chest X-ray and laboratory tests were unremarkable. Transthoracic echocardiography demonstrated a right coronary artery-right ventricular fistula. Computed tomography and three-dimensional coronary artery computed tomography angiography (Supplementary Video 1) showed the left and right main coronary artery, the left anterior descending artery and the circumflex artery were enlarged and tortuous. There is an interruption between right sinus of Valsalva and the right main coronary artery (Figure 1A, arrow), single left coronary ostium with a giant coronary trunk (Figure 1B, arrow). A branch from the proximal end of right coronary artreatery was inserted into the right ventricle through a 7-mm fistula (Figure 1C, arrow). The right main coronary artery was connected with the left conus branch via the collateral branch while the left circumflex was communicated with the right coronary artery thus forming a closed loop at the base of the heart (Figure 1D). Ascending aortic angiography was performed and further confirmed this malformation (Figure 2, Supplementary Video 2). There was no other notable medical or surgical history and no family history of cardiovascular disease. Because of extreme vessel tortuosity and inability to deliver a catheter far enough distally, the coronary fistula was then surgically ligated with off-pump strategy (Figure 3). The patient discharged on postoperative day 5 and free of symptoms during the 3 years of follow-up (Figure 4).

## Discussion and Conclusions

Single coronary artery (SCA) is defined as an isolated coronary artery that arises from a single coronary ostium (with or without a coronary artery trunk) and provides blood supply to the entire myocardium (1). SCA could be classified into two main categories: left type (L) or right type (R) according to the origin location and anatomical distribution of branches. The categories were then designated as group I, II, or III according to the anatomical course. Group I had an anatomical course of either a RCA or LCA. Group II anomalies originate from the proximal part of the normal RCA or LCA, and cross the base of the heart before reaching the normal site of the inherent coronary artery. Group III specifically indicates the SCA arising from the right sinus of valsalva, with the left anterior descending (LAD) and left circumflex (LCx) branch originating separately from a common trunk (6). According to this classification, our patient presented with an L-II type of SCA originating from the left aortic sinus, and the right coronary artery was connected to the left conus branch via the lateral branch. It is important to know the origin and distribution of the whole coronary artery. In the presented case, the left circumflex was communicated with the right coronary artery thus a closed loop was formed at the base of the heart.

SCA can either be isolated or coexist with other congenital heart anomalies such as coronary arteriovenous fistula (3, 5), coronary aneurysm (7), patent foramen ovale (8), interventricular septal defect (9), patent ductus arteriosus (10), tetralogy of Fallot (11), transposition of great vessels (12), truncus arteriosus (13), and bicuspid aortic valve (14). CAF is a rare condition in which the coronary arteries communicate directly with the heart chambers or vessels. Selective angiography allows detection of SCA associated with CAF in most patients in the early stage (5). Preoperative multi-detector computed tomography (MDCT) coronary angiography, due to the non-invasive detection and the ability to reveal complex coronary artery anatomy, is recommended by the American Heart Association Committee to characterize these anomalies in conjunction with invasive coronary angiography (15, 16).

Most patients with CAF are asymptomatic at the early stage, but elderly patients may present with exertional chest pain, fatigue, palpitations, arrhythmias, congestive heart failure, or possibly sudden death (16, 17). CAF may result in preferential blood flow from coronary circulation to pulmonary circulation, leading to the coronary-steal-related chronic myocardial ischemia (17). Vieussens' arterial ring (VAR) is a rare anatomic variant and refers to the collateral pathway between the conus branches of the right and left coronary arterie that was first described by Raymond de Vieussens (18). Four subgroups of VAR were found and defined as a collateral pathway originating from the right anterior conus artery, a collateral pathway passing in front of the pulmonary artery, a collateral pathway connecting the right and a collateral pathway connecting the left coronary artery circulation: VAR accompanying with and without vascular pathology like aneurysm or fistula (Type 1B and 1A, respectively), with a short LAD branch (Type 2) and with a single coronary artery anomaly (Type 3) (19). In the presented case, the right main coronary artery was connected with the left conus branch via the collateral branch while the left circumflex was communicated with the right coronary artery thus forming a closed loop at the base of the heart (Figure 1D), which was unlike the subtypes of VAR and was a previously unreported anatomy. Thus, we named it the congenital coronary artery ring or Yang's ring. However, there are no treatment guidelines or follow-up recommendations currently. Management of these anomalies remains controversial, especially in asymptomatic patients. Various treatment modalities such as coil embolization, catheter-mediated stent occlusion, and surgical ligation are available (17). Catheter techniques are difficult or impossible in a small percentage of patients, due to extreme vessel tortuosity and inability to deliver a catheter far enough distally (20). Because of the high rate of late symptoms and complications, especially when the shunt is significant (Qp/Qs ratio > 1.5), early surgery is an optimal treatment in the case of SCA combined with CAF (5, 21). In the present case, the vessels are extreme tortuous and inable to deliver a catheter far enough through the coronary truck. Thus, a surgically ligated with off-pump strategy was recommended.

## Data Availability Statement

The raw data supporting the conclusions of this article will be made available by the authors, without undue reservation.

## Ethics Statement

The study protocol was approved by the Ethics Committee of the Second Xiangya Hospital of Central South University, Changsha, China. Written informed consent to participate in this study was provided by the participants' legal guardian/next of kin.

## Author Contributions

ST and CF drafted the manuscript. CF and JY designed the study. CD, MT, and JY revised the manuscript. ST, MT, and JY were responsible for the collection of data or analysis. All authors read and approved the final manuscript.

## Conflict of Interest

The authors declare that the research was conducted in the absence of any commercial or financial relationships that could be construed as a potential conflict of interest.

## Publisher's Note

All claims expressed in this article are solely those of the authors and do not necessarily represent those of their affiliated organizations, or those of the publisher, the editors and the reviewers. Any product that may be evaluated in this article, or claim that may be made by its manufacturer, is not guaranteed or endorsed by the publisher.

## Supplementary Material

Supplementary Video 1 Three-dimensional coronary artery computed tomography angiography: A giant coronary trunk from left coronary sinus then divide into two main branches. The left and right main coronary artery, the left anterior descending artery and the circumflex artery were enlarged and tortuous. A branch from the proximal end of right coronary artery was inserted into the right ventricle through a 7-mm fistula. The right main coronary artery was connected with the left conus branch via the collateral branch while the left circumflex was communicated with the right coronary artery thus forming a closed loop.

Supplementary Video 2 Ascending aortic angiography: Confirming the diagnosis of single left coronary ostium with a giant coronary trunk, right coronary artery-right ventricular fistula and a coronary artery ring.

## Abbreviations

CAF: Coronary artery fistula
CAR: Coronary artery ring
LAD: Left anterior descending
LCx: Left circumflex
MDCT: Multi-detector computed tomography
RV: Right ventricle
SCA: Single coronary artery.
